# Food allergy and PPI-responsive esophageal eosinophilia

**DOI:** 10.1186/1710-1492-10-S2-A25

**Published:** 2014-12-18

**Authors:** Jason K Ko, David JT Huang, Jorge A Mazza

**Affiliations:** 1 Schulich School of Medicine & Dentistry, University of Western Ontario, London, Ontario, Canada; 2 Division of Allergy & Clinical Immunology, Department of Medicine, University of Western Ontario, London, Ontario, Canada

## Background

Eosinophilic esophagitis (EoE) is considered a chronic condition mediated by immune reaction to food and/or environmental allergens. Though first recognized decades ago, the characterization of EoE is ongoing and an important aspect of this process is the distinction between EoE and other forms of esophageal eosinophilia [1,2]. One group of patients exhibit marked esophageal eosinophilia (>15 eo/HPF), negative esophageal pH-monitoring studies and yet have clinicopathologic response to proton-pump inhibitor (PPI) treatment: this group is categorized as having PPI-responsive esophageal eosinophilia (PPI-REE) [1,3]. It is not certain whether those with PPI-REE are cases of GERD undiagnosed by pH-monitoring, EoE responding to PPI therapy as in-vitro studies suggest [4], or some combination thereof. GERD is orders of magnitudes more prevalent than EoE and thus misdiagnosed cases of GERD could have significant impact on any study of EoE patients [5,6]. Other groups have attempted to distinguish cases of PPI-REE and EoE, but failed to do so using clinicopathologic criteria [7,8]. This retrospective review of patients diagnosed with EoE aimed to differentiate PPI-REE and non-responsive patients, with an emphasis on prevalence of food allergy between the two groups.

## Methods

A chart review was performed for 30 patients diagnosed with EoE, prescribed PPI therapy and tested with atopic patch tests for a panel of food allergens. Patients were categorized as having PPI-REE if past clinical assessments noted significant symptomatic improvement with PPI therapy.

## Results

Of the 30 patients reviewed, 12 were found to have PPI-REE. There was no significant difference in other treatments offered to PPI-REE and non-responsive patients, the eosinophil counts at diagnosis, nor in likelihood of food allergy as detected by skin prick or food patch testing (Table 1).

**Table 1:** Characteristics of PPI-REE and non-responder groups.

	PPI-REE	Non-responders	p-value
***N***	12	18	N/A

**Average eosinophil count at diagnosis (eo/HPF)**	65.7 ± 29.2	42.6 ± 15.6	0.14

**Use of other treatments (Swallowed steroid and/or dilatation)**	10/12 (83%)	8/18 (44%)	0.21

**Food allergy on atopic patch test**	9/12 (75%)	9/18 (50%)	0.60

**Food allergy on skin prick test**	5/11 (45%)	6/16 (38%)	0.98

## Conclusions

It was hypothesized that PPI-REE cases would be less atopic, with regards to foods, than non-responders due to the possible prevalence of undiagnosed GERD in the former group. However, this review failed to show any statistically significant differences between the two groups. This is consistent with attempts of other groups to distinguish PPI-REE and EoE patients.
